# Conservatively managed patients with non-ST-segment elevation acute coronary syndrome are undertreated with indicated medicines

**DOI:** 10.1371/journal.pone.0208069

**Published:** 2018-11-28

**Authors:** Elena Candela, Francisco Marín, José Miguel Rivera-Caravaca, Nuria Vicente Ibarra, Luna Carrillo, María Asunción Esteve-Pastor, Teresa Lozano, Manuel Jesús Macías, Vicente Pernias, Miriam Sandín, Esteban Orenes-Piñero, Miriam Quintana-Giner, Ignacio Hortelano, Beatriz Villamía, Andrea Veliz, Mariano Valdés, Juan G. Martínez-Martínez, Juan M. Ruiz-Nodar

**Affiliations:** 1 Department of Cardiology, Instituto de Investigación Sanitaria y Biomédica de Alicante (ISABIAL), Hospital General Universitario de Alicante, Alicante, Spain; 2 Department of Cardiology, IMIB-Arrixaca CIBER-CV, Hospital Clínico Universitario Virgen de la Arrixaca, Murcia, Spain; 3 Department of Cardiology, Hospital General Universitario de Elche, Alicante, Spain; San Giovanni Addolorata Hospital, ITALY

## Abstract

**Introduction and aims:**

Patients with non-ST-elevation acute coronary syndrome (NSTE-ACS) are often managed conservatively. Clinical practice guidelines recommend treating these patients with the same pharmacological drugs as those who receive invasive treatment. We analyze the use of new antiplatelet drugs (NADs) and other recommended treatments in people discharged following an NSTE-ACS according to the treatment strategy used, comparing the medium-term prognosis between groups.

**Methods:**

Prospective observational multicenter registry study in 1717 patients discharged from hospital following an ACS; 1143 patients had experienced an NSTE-ACS. We analyzed groups receiving the following treatment: No cardiac catheterization (NO CATH): n = 134; 11.7%; Cardiac catheterization without revascularization (CATH-NO REVASC): n = 256; 22.4%; percutaneous coronary intervention (PCI): n = 629; 55.0%; and coronary artery bypass graft (CABG): n = 124; 10.8%. We assessed major adverse cardiovascular events (MACE), all-cause mortality, and hemorrhagic complications at one year.

**Results:**

NO CATH was the oldest, had the most comorbidities, and was at the highest risk for ischemic and hemorrhagic events. Few patients who were not revascularized with PCI received NADs (NO CATH: 3.7%; CATH-NO REVASC: 10.6%; PCI: 43.2%; CABG: 3.2%; p<0.001). Non-revascularized patients also received fewer beta-blockers, angiotensin-converting enzyme (ACE) inhibitors, angiotensin II receptor blockers (ARB), and statins (p<0.001). At one year, MACE incidence in NO CATH group was three times that of the other groups (30.1%, p<0.001), and all-cause mortality was also much higher (26.3%, p<0.001). There were no significant differences in hemorrhagic events. Belonging to NO CATH group was an independent predictor for MACE at one year in the multivariate analysis (HR 2.72, 95% CI 1.29–5.73; p = 0.008).

**Conclusions:**

Despite current invasive management of NSTE-ACS, patients not receiving catheterization are at very high risk for under treatment with recommended drugs, including NADs. Their medium-term prognosis is poor, with high mortality. Patients treated with PCI receive better pharmacological management, with high use of NADs.

## Introduction

Clinical practice guidelines for non-ST-elevation acute coronary syndrome (NSTE-ACS) recommend a standard invasive strategy, consisting of coronary catheterization followed by coronary revascularization [1,2]. However, about 45%-60% of patients with NSTE-ACS only receive conservative medical treatment [3,4].

Several studies report that the population who does not undergo coronary catheterization is a high-risk group, with higher comorbidities and incidence of cardiovascular events in the medium and long term [3,4]. Despite this high risk, different studies have shown that patients who receive medical treatment alone are often undertreated pharmacologically, i.e., a lower proportion of these patients followed the optimal recommended drug treatment compared to those who are revascularized [5,6].

The CURE study found that at one-year follow-up, the NSTE-ACS patients receiving dual antiplatelet therapy (DAPT, aspirin plus clopidogrel) presented lower combined rates of cardiovascular death, myocardial infarction, and stroke compared to those taking aspirin alone [7]. This reduction in cardiovascular risk was similar in patients treated medically and in those who received revascularization therapy. Nevertheless, while most patients who undergo an angioplasty continue their treatment with DAPT, less than half of those treated medically are discharged with a second antiplatelet agent, even though both groups can achieve similar benefits [3].

Moreover, new antiplatelet drugs (NADs) such as ticagrelor have shown better outcomes than older agents like clopidogrel for improving the prognosis of patients managed medically following acute coronary syndrome (ACS) [7,8]. In light of this new evidence, updated clinical practice guidelines recommend ticagrelor over clopidogrel following ACS, regardless of the initial treatment strategy [1,2,9].

The objective of this study is to assess the use of NADs and other recommended treatments in patients discharged following an NSTE-ACS according to the initial treatment strategy used. We also evaluate the medium-term prognosis and its association with different inpatient treatment strategies.

## Materials and methods

### Study design

The rationale and design of this study have been described previously [10]. In brief, this is a prospective observational multicenter study, including all patients consecutively discharged with definitive diagnosis of ACS in three tertiary hospitals for a period of nearly two years (from February 1, 2014 to December 31, 2015). Only those patients who died during hospitalization or experienced an ACS during another extracardiac pathological condition (stroke, sepsis, surgery, or trauma) were excluded, without other specific exclusion criteria. They signed the informed consent of the registry, without the non-signature of consent implying a change in their therapeutic approach, hospital management or follow-up.

Our study protocol complies with the Helsinki Declaration and was approved by the Ethical Research and Reference Committee after being accepted by the Department of Medicine for Human Use from the National Agency for Medicines and Medical Devices, with reference JRN-NAG-2014-01.

An independent clinical research organization (CRO) performed an external audit of the registry, evaluating the appropriateness of patient inclusion and the accuracy of the data in all participating hospitals, as well as the possible existence of eligible patients who may have been excluded during the recruitment period.

### Study population

Of the 1717 patients included in the study, we analyzed 1143 who were discharged following a diagnosis of NSTE-ACS (including both non-ST-elevation myocardial infarction [NSTEMI] and unstable angina). We assessed the use of different therapies recommended by clinical practice guidelines according to the treatment that patients received during hospitalization. The four treatment categories were coded as follows: 1-NO CATH: medical treatment without catheterization; 2-CATH-NO REVASC: catheterization without revascularization; 3-PCI: percutaneous coronary intervention; 4-CABG: coronary artery bypass graft.

We analyzed the use of antiplatelet drugs during hospital stay and upon discharge, comparing prescriptions for the traditional agent, clopidogrel, versus prescriptions for NADs. We also evaluated the use of other drugs indicated for managing NSTE-ACS, such as beta-blockers, angiotensin-converting enzyme (ACE) inhibitors, angiotensin II receptor blockers (ARBs), and statins.

We recorded patients’ baseline clinical characteristics and carried out clinical follow-up (by phone or by consulting patients’ electronic medical records) at three months and one year. During this period we recorded any major adverse cardiovascular event (MACE), defined as cardiovascular death, non-fatal myocardial infarction, or non-fatal ischemic stroke. We also collected data on all-cause mortality and hemorrhagic complications according to the Bleeding Academic Research Consortium (BARC) definitions [11] and the thrombolysis in myocardial infarction (TIMI) criteria [12]. Bleeding was described as: all-cause hemorrhage (BARC 1–5), clinically relevant hemorrhages (BARC 2–5), and major hemorrhages (BARC 3–5). Major bleeding according to the TIMI criteria was defined as any type of intracranial bleeding, signs of clinically evident hemorrhage associated with a fall in hemoglobin of more than 5 g/dL, or fatal bleeding.

### Statistical analysis

We expressed categorical variables as frequencies (percentages), comparing them by the χ^2^statistic. Continuous variables were expressed as mean (standard deviation [SD]) if normally distributed and as median (interquartile range) if the distribution was non-parametric. We used analysis of variance (ANOVA) to compare groups by continuous variables and the Kruskal-Wallis test if the distribution was not normal.

We performed Cox regression models (with hazard ratios [HRs] and 95% confidence intervals [CIs]) to determine which variables showed an independent association at one year follow-up with MACE, all-cause mortality, or major hemorrhage (BARC ≥3).

We considered p values <0.05 as statistical significant for all tests performed. We used SPSS statistical software (version 20.0, SPSS Inc., Chicago, Illinois, USA) for all analyses. The authors of this registry study are solely responsible for the study design, analysis, and development of this paper.

## Results

We included 1143 patients with NSTE-ACS. Mean age was 68±12.7 years, and 69.1% were men. At discharge, 298 (26.1%) patients had unstable angina and 845 (73.9%) had NSTEMI. Most patients (n = 1009; 88.3%) underwent catheterization during hospitalization.

**Fig 1:**
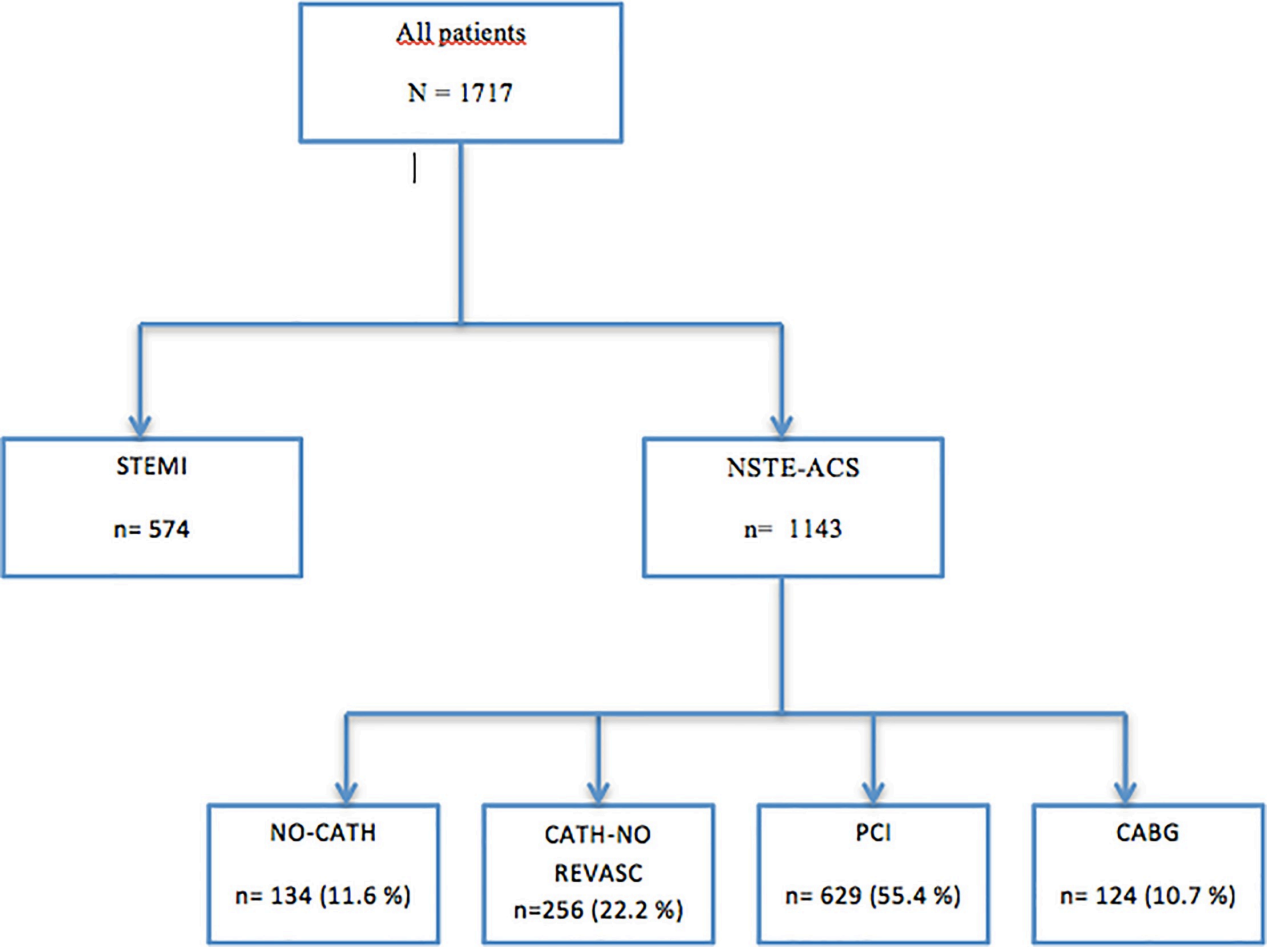
Flow chart of patients and analyzed subgroups. STEMI: ST-elevation myocardial infarction; NSTE-ACS: non-ST-elevation ACS; PCI: percutaneous coronary intervention; CABG: coronary artery bypass graft.

The distribution of patients by treatment groups was as follows: 1-NO CATH, 134 (11.7%) patients; 2-CATH-NO REVASC: 256 (22.4%) patients; 3-PCI: 629 (55.0%) patients; 4-CABG: 124 (10.8%) patients (Fig 1). The main reasons for not performing catheterization in group 1 were: high comorbidity (30.1%), known coronary anatomy (28.6%), and elderly age (17.5%). In 51.6% of the patients from group 2 (CATH-NO REVASC), catheterizations showed no significant coronary lesions.

### Baseline clinical characteristics and form of presentation

Mean age in the NO-CATH group was significantly higher (p<0.001) than in the rest of groups (Table 1). Moreover, this group had a higher prevalence of cardiovascular risk factors and comorbidities, and they were at higher risk for ischemic and hemorrhagic complications assessed by the GRACE and CRUSADE scores, respectively. Group 3 (PCI) stands out for the relatively low proportion of women and the high proportion of smokers. About 10% of the overall study population had a history of atrial fibrillation, with the higher proportion occurring in the NO-CATH group. In terms of how NSTE-ACS is presented, unstable angina was comparable across all groups (p = 0.362).

**Table 1:** Baseline patient characteristics.

	Treatment category (N = 1143)				
	NO CATH n = 134 (11.7%)	CATH-NO REVASC n = 256 (22.4%)	PCI n = 629 (55.0%)	CABG n = 124 (10.8%)	p value
**Age in years**	79.4±10.6	67.8 ± 12.4	65.6 ±12.4	67.8 ± 9.5	<0.001
**Women**	55 (41.0%)	110 (43.0%)	157(25.0%)	21 (25.0%)	<0.001
**Hypertension**	113 (84.3%)	203 (79.3%)	427 (68.0%)	89 (71.8%)	<0.001
**Dyslipidemia**	84 (62.7%)	164 (64.1%)	391 (62.2%)	89 (71.8%)	<0.001
**Diabetes mellitus**	71 (53.0%)	98 (38.3%)	257 (40.9%)	63 (50.8%)	<0.001
**Current smoker**	19 (14.3%)	71 (27.7%)	228 (36.3%)	36 (29.0%)	<0.001
**Previous coronary disease**	70 (52.2%)	83 (32.4%)	190 (30.2%)	36 (29.0%)	<0.001
**Peripheral arterial disease**	25 (18.7%)	26 (10.2%)	55 (8.7%)	17 (13.7%)	0.006
**Stroke**	27 (20.1%)	25 (9.8%)	55 (8.7%)	10 (8.1%)	0.001
**Atrial fibrillation**	24 (18.2%)	34 (13.3%)	51 (8.1%)	6 (4.8%)	0.002
**Prior aspirin**	77 (57.5%)	102 (39.8%)	252 (40.1%)	57 (46.0%)	0.002
**Prior clopidogrel**	37 (27.6%)	45 (17.6%)	92 (14.6%)	21 (16.9%)	0.004
**Prior anticoagulation**	16 (11.9%)	38 (14.8%)	59 (9.4%)	6 (4.8%)	0.014
**Body mass index (kg/m**^**2**^**)**	27.3 ±4.8	28.8 ±5.2	28.8 ±4.5	27.8 ±4.1	0.008
**Characteristics of presentation**					
*Troponin positive*	105 (78.4%)	182 (71.1%)	470 (74.7%)	88 (71.0%)	0.362
*LVEF*, *%*	56.6 ±12.5	57.3 ±10.9	57.8 ±10.3	57.4 ±12.5	0.677
*eGFR*, *ml/min/1*.*73 m*^*2*^	56.8 ± 30.1	77.5 ± 27.8	78.3 ±28.6	77.4 ±24.9	<0.001
**GRACE risk score**	161.6 ±41.8	128.7 ±38.6	124.5 ±39.9	130.8 ±36.6	<0.001
**CRUSADE risk score**	45.0 ±19.1	31.6 ±14.2	28.2 ±15.2	29.9 ±14.1	<0.001

Data presented as n (%) or mean ± standard deviation.

LVEF: left ventricular ejection fraction; eGFR: estimated glomerular filtration rate; GRACE: Global Registry of Acute Coronary Events.

### Differences in pharmacological treatment during admission and upon discharge

Although the use of aspirin was similar across groups, patients from group 1 received less P2Y_12_ inhibitors in the first 24 hours of admission, and they also showed less use of beta-blockers, ACE inhibitors/ARBs, and statins (Table 2).

**Table 2:** Management during admission.

	Treatment category (N = 1134)				
	NO CATH n = 134 (11.7%)	CATH-NO REVASC n = 256 (22.4%)	PCI n = 629 (55.0%)	CABG n = 124 (10.8%)	p value
**Coronary angiography performed**	0 (0%)	256 (100%)	629 (100%)	124 (100%)	<0.001
**Acute treatment (<24h)**					
*Aspirin*	129 (96.3%)	252 (98.4%)	623 (99.0%)	123 (99.2%)	0.088
*Heparin*	103 (76.9%)	214 (83.6%)	504 (80.5%)	94 (75.8%)	0.026
*Clopidogrel*	96 (71.6%)	192 (75.0%)	490 (77.9%)	84 (67.7%)	<0.001
*Prasugrel*	1 (0.7%)	2 (0.8%)	20 (3.2%)	1 (0.8%)	<0.001
*Ticagrelor*	8 (6.0%)	32 (12.5%)	114 (18.1%)	21 (16.9%)	<0.001
*Beta-blockers*	98 (73.1%)	208 (81.3%)	535 (85.1%)	108 (87.1%)	0.004
*ACE inhibitors/ARB*	94 (70.1%)	223 (87.1%)	544 (86.5%)	102 (82.3%)	<0.001
*Statins*	122 (91.0%)	242 (94.5%)	607 (96.5%)	123 (99.2%)	0.006
**Coronary disease**					
*No significant lesions*		132 (51.6%)	0	0	
*3 vessels or left main*		44 (17.2%)	148 (23.5%)	110 (94.9%)	<0.001
**Complete revascularization**			437 (69.5%)	107 (89.2%)	<0.001
**Use of DES**			488 (78.6%)		

Data presented as n (%).

ACEI: angiotensin converting enzyme inhibitors; ARB: angiotensin II receptor blockers; DES: drug-eluting stent.

Upon discharge, the use of P2Y_12_ inhibitors and dual antiplatelet therapy was much higher in the PCI group (99.4% and 99.5% respectively) compared to the other groups (p<0.001) (Table 3). Those treated with PCI also received significantly more NADs (43.2%) than the rest of the patients (NO-CATH 3.7%, CATH-NO REVASC 10.6%, CABG 3.2%; p<0.001), while it is true that prasugrel is contraindicated in patients in whom coronary anatomy is unknown and this should be taken into account when interpreting this results. With regard to the rest of the treatments prescribed at discharge (aspirin, beta-blockers, ACE inhibitors/ARBs, and statins), their use was significantly lower in the two groups (1 and 2) managed medically.

**Table 3:** Treatment at discharge.

	Treatment category (N = 1143)				
	NO CATH n = 134 (11.7%)	CATH-NO REVASC n = 256 (22.4%)	PCI n = 629 (55.0%)	CABG n = 124 (10.8%)	p value
**Aspirin**	120 (89.6%)	227 (88.7%)	624 (99.2%)	115 (92.7%)	<0.001
**P2Y**_**12**_**-receptor inhibitor**					
*Any P2Y*_*12*_	80 (59.7%)	155 (60.6%)	625 (99.4%)	43 (34.7%)	<0.001
*Clopidogrel*	75 (56.0%)	128 (50.0%)	353 (56.1%)	39 (31.5%)	<0.001
*Prasugrel*	0	1 (0.4%)	55 (8.7%)	0	<0.001
*Ticagrelor*	5 (3.7%)	26 (10.2%)	217 (34.5%)	4 (3.2%)	<0.001
*Dual antiplatelet therapy*	87 (64.9%)	178 (69.5%)	626 (99.5%)	46 (37.1%)	<0.001
**Beta-blockers**	92 (69.2%)	187 (73.0%)	528 (83.9%)	111 (89.5%)	<0.001
**ACEI or ARB**	90 (67.2%)	202 (78.9%)	521 (82.8%)	79 (63.7%)	<0.001
**Statins**	122 (91.0%)	229 (89.5%)	608 (96.7%)	119 (96.7%)	<0.001
**Oral anticoagulant**	24 (17.9%)	47 (18.4%)	74 (11.8%)	14 (11.3%)	0.191

Data are represented as total (%).

ACEI: angiotensin converting enzyme inhibitors; ARB: angiotensin II receptor blockers.

### One-year follow-up

One-year follow-up was achieved in 98.25% of the sample. In our registry, the NO-CATH group presented the worst prognosis, with three times higher incidence of MACE compared to the other groups (Table 4). All-cause mortality was also more frequent in this group (26.3%) and the main causes of non-cardiovascular death were, in this order, oncologic diseases, sepsis and hemorrhages. Of note, almost half of deaths were by a non-cardiovascular cause. In the other groups, cardiovascular mortality was lower than 3%. Stroke incidence was highest in the surgical group (3.3%; p<0.001).

**Table 4:** Medium-term events.

	Treatment category (N = 1123)				
	NO CATH n = 133 (11.8%)	CATH-NO REVASC n = 254 (22.6%)	PCI n = 613 (54.6%)	CABG n = 123(11.0%)	p value
**Ischemic events**					
***Total MACE***	40 (30.1%)	20 (7.9%)	64 (10.4%)	11 (8.9%)	<0.001
*Cardiovascular death*	18 (13.5%)	7 (2.8%)	14 (2.3%)	2 (1.6%)	<0.001
*Non-fatal myocardial infarction*	22 (16.5%)	9 (3.5%)	41 (6.7%)	5 (4.1%)	<0.001
*Non-fatal stroke*	0	4 (1.6%)	9 (1.5%)	4 (3.3%)	<0.001
**Death from any cause**	35 (26.3%)	11 (4.3%)	22 (3.6%)	5 (4.1%)	<0.001
**Bleeding events**					
*BARC 1–5*	17 (12.8%)	21 (8.3%)	72 (11.7%)	10 (8.1%)	0.292
*BARC 2–5*	17 (12.8%)	20 (7.9%)	54 (8.8%)	10 (8.1%)	0.414
*BARC ≥3*	4 (3.0%)	6 (2.4%)	18 (2.9%)	5 (4.1%)	0.839
*TIMI major*	3 (2.3%)	3 (1.2%)	9 (1.5%)	3 (2.4%)	0.738

MACE: major adverse cardiovascular events (cardiovascular death, stroke and acute non-fatal myocardial infarction); BARC: Bleeding Academic Research Consortium; TIMI: thrombolysis in myocardial infarction

With regard to the incidence of hemorrhagic events within a year of discharge, we did not observe significant differences between groups in total hemorrhages or in the most serious hemorrhages (major hemorrhage according to TIMI criteria and BARC ≥ 3).

**Fig 2:**
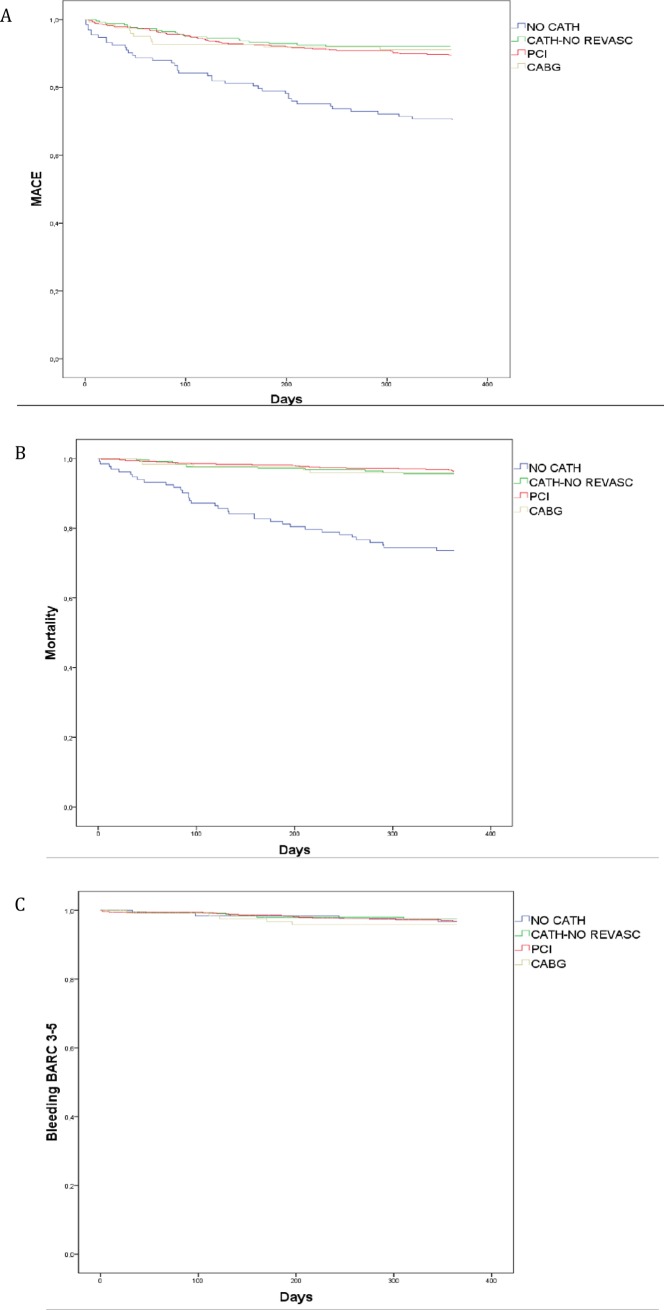
Kaplan-Meier survival curves in the four predefined subgroups. A: Kaplan-Meier survival curve for major adverse cardiovascular events (MACE: cardiovascular death, stroke and acute non-fatal myocardial infarction). Log-Rank test (Mantel-Cox); p<0.001. B: Kaplan Meier survival curve for all-cause mortality. Log-Rank test (Mantel-Cox); p<0.001. C: Kaplan Meier survival curve for BARC 3–5 bleeding events. Log-Rank test (Mantel-Cox); p = 0.846.

Fig 2 shows the survival curves for MACE, all-cause mortality, and major hemorrhage (BARC ≥3).

Cox regression analysis (Table 5) identified the following variables as independent predictors for presenting MACE at one-year follow-up: peripheral artery disease (HR 1.70, 95% CI 1.05, 2.73; p = 0.030), the Killip-Kimball classification (HR 1.43, 95% CI 1.08–1.89; p = 0.014), and the absence of catheterization (HR 2.72, 95% CI 1.29–5.73; p = 0.008). We also observed independent associations between all-cause mortality and both the level of hemoglobin (HR 0.87, 95% CI 0.76–0.99; p = 0.03) and Killip-Kimball classification (HR 1.71, 95% CI 1.21–2.42; p = 0.003), while non-performance of catheterization showed a trend to significance.

**Table 5:** Independent predictors of adverse events during follow-up by Cox regression analysis.

Events	Variables	Univariate analysis HR (95% CI); p	Multivariate analysis HR (95% CI); p
**MACE**^**a**^			
	*Age*	1.03 (1.02–1.05); <0.001	
	*Hypertension*	1.94 (1.23–3.07); 0.002	
	*Diabetes mellitus*	1.66 (1.18–2.32); 0.003	
	*Peripheral arterial disease*	2.54 (1.69–3.81); <0.001	1.70 (1.05, 2.73); 0.030
	*Stroke*	2.09 (1.36–3.24); 0.002	
	*Haemoglobin*	0.85 (0.79–0.91); <0.001	
	*eGFR*	0.99 (0.98–0.99); <0.001	
	*Killip class*	1.62 (1.29–2.02); <0.001	1.43 (1.08, 1.89); 0.014
	*No catheterization (Group 1)*	3.74 (1.92–2.02); <0.001	2.72 (1.29, 5.73);0.008
**All-cause mortality**			
	*Age*	1.08 (1.06–1.11); <0.001	
	*Hypertension*	3.08 (1.48–6.41); <0.001	
	*Diabetes mellitus*	1.55 (0.98–2.45); 0.062	
	*Peripheral arterial disease*	2.59 (1.51–4.47); 0.002	
	*Stroke*	3.03 (1.78–5.16); <0.001	
	*Haemoglobin*	0.71 (0.65–0.78); <0.001	0.87 (0.76, 0.99); 0.038
	*eGFR*^*b*^, *ml/min/1*.*73 m*^*2*^	0.97 (0.96–0.98); <0.001	
	*Killip class*	2.15 (1.67–2.76); <0.001	1.71 (1.21, 2.42); 0.003
	*No catheterization (Group 1)*	7.37 (2.89–18.82); <0.001	2.87 (0.98, 8.39); 0.054
**Hemorrhage BARC**^**c**^ **3–5**			
	*Age*	1.05 (1.02–1.08); <0.001	
	*Hypertension*	2.76 (0.96–7.76); 0.033	
	*Diabetes mellitus*	0.85 (0.42–1.72); 0.659	
	*Peripheral arterial disease*	1.51 (0.58–3.92); 0.415	
	*Stroke*	2.05 (0.85–4.97); 0.141	
	*Haemoglobin*	0.70 (0.61–0.81); <0.001	0.74 (0.61, 0.89): 0.002
	*eGFR*	0.98 (0.97–0.99); 0.009	
	*Killip class*	1.39 (0.86–2.25): 0.210	
	*No catheterization (Group 1)*	0.77 (0.21–2.86); 0.851	

MACE^a^: cardiovascular death, stroke and acute nonfatal myocardial infarction; eGFR^b^: estimated glomerular filtration rate; BARC^c^: Bleeding Academic Research Consortium.

In addition, hemoglobin was the only variable showing to be an independent risk factor for major hemorrhage (BARC ≥3) at one year (HR 0.74, 95% CI 0.61–0.89; p = 0.002). This outcome was not associated with the use of NADs.

## Discussion

In this contemporary registry, our data suggest that: (i) patients with NSTE-ACS who do not undergo catheterization are a high-risk population undertreated with NADs and other recommended drugs; (ii) this group also has a poor prognosis in the medium term, with an elevated incidence of MACE and high mortality, although a similar prevalence of hemorrhagic events to other NSTE-ACS patients; (iii) optimal medical treatment and prescription of NADs takes place primarily in patients treated with PCI.

In our registry, patients managed with medical treatment alone constitute a third of all those diagnosed with NSTE-ACS. In agreement with most similar studies [3,6] the proportion of this population who receives conservative treatment is still high in ACS, despite the dramatic rise in the use of percutaneous revascularization techniques. However, these patients have a great heterogeneity, and their presence in large clinical trials is scarce, what makes ‘real world’ registries like ours clinically useful.

As other authors have previously noted [3,4,6], the present study shows that this patient group receive treatments recommended by clinical practice guidelines in a lower proportion than other NSTE-ACS patients. Although these results are quite far from ideal, there are data suggesting that clinical practice in this area has been improving in recent years, with a trend toward optimizing pharmacological treatment for ACS that has shown the most notable benefits in patients managed conservatively, albeit pharmacological treatment strategies in this group are still suboptimal compared to patients treated with PCI [5].

Although double antiplatelet therapy is supported by a class I recommendation [1,2,9], with studies showing similar efficacy regardless of the therapeutic strategy chosen [8,13,14], patients managed conservatively show conspicuously low prescription rates for P2Y_12_ inhibitors at discharge—just 60%—while virtually all patients who undergo revascularization with PCI receive them. In line with this data, a previous large controlled study demonstrated that less than half of the conservatively managed patients received clopidogrel at discharge [15].

In this study, in patients who did not undergo catheterization, the most commonly used antiplatelet agent was clopidogrel. Thus, it seems that NADs is basically reserved for patients treated with PCI, even though numerous studies have shown comparable benefits across treatment groups [8, 9, 13, 16]. Indeed, in the PLATO study [8], the subgroup of conservatively managed participants (28% of the total) showed lower incidence of the primary outcome (cardiovascular death, myocardial infarction, and stroke) when taking ticagrelor versus clopidogrel (12% *vs*. 14.3%; HR 0.85, 95% CI 0.73–1.00; p = 0.04), with similar results for all-cause mortality (6.1% *vs*. 8.2%; HR 0.75, 95% CI 0.61–0.93; p = 0.01) and without a significant increase in major bleeding.

Clinicians’ reluctance to use more potent antiplatelet drugs in this group may be partly due to the higher prevalence of comorbidities, advanced age, history of stroke and hemorrhagic risk in this population, which makes difficult to discern when the risks outweigh the potential benefits of these drugs. Conservatively managed patients are also prescribed the highest relative proportion of anticoagulants at discharge (17.9%), which is a contraindication for NADs.

### Medium-term prognosis

With regard to medium-term prognosis, and in agreement with most registries published to date [6], patients selected for medical treatment without catheterization in our cohort was a high-risk population, showing much higher mortality rates due to both cardiovascular and other causes as well as a higher risk of ischemic events. In part, the baseline characteristics of these patients can explain these outcomes: older age, higher comorbidities, and higher history of coronary and peripheral artery diseases. These patients showed a higher ischemic risk according to the GRACE score and higher hemorrhagic risk according to the CRUSADE. However, another factor that could be related to these patients’ poor prognosis in the medium is the low use of drugs indicated for NSTE-ACS, despite that DAPT, has of the most relative benefit in people >70 [3]. Thus, the underuse of treatments with proven benefits for preventing ischemic events probably plays an important role in the prognosis of these patients [15].

Additionally, and despite the higher baseline bleeding risk in this elderly subgroup, the group of patients managed conservatively did not show significant differences with other groups in terms of total or major hemorrhages. Although the low use of DAPT could be the reason for these results, it is also true that this population showed the highest use of oral anticoagulants at discharge. Thus, ischemic events determine the poor prognosis, as is shown graphically in the survival curves.

While patients who do not undergo catheterization are at the highest risk for harmful outcomes and have the worst prognosis, they are also the most undertreated group, especially with regard to DAPT. Thus, this subgroup is the main target for improved clinical management, even if their overall clinical profile makes unlikely that the rates of optimal treatment in this group remain optimal. There is a need for more real-world data emphasizing the potential benefits of DAPT and especially NADs for the prognosis of these patients [16].

With regard to the type of revascularization, both techniques resulted in a similar prognosis. The presence of multivessel or left main disease is the main determinant of surgical revascularization. Antiplatelets are also underused in patients treated with CABG, who receive less aspirin than those treated with PCI and lower prescription rates of P2Y_12_ inhibitors (34.7%). CABG provides more complete revascularization than PCI (89.2% *vs*. 69.6%; p<0.001), although in our cohort both groups presented similar ischemic and hemorrhagic prognoses in the medium term. Of note, incidence of stroke in the CABG group was more than double than one of the PCI group (3.3% *vs*. 1.5%; p<0.001), which is consistent with the results of the SYNTAX trial, showing a significant trend toward higher incidence of stroke in surgical patients (3.7% *vs* 2.4%; p = 0.09) [17].

Among the predictive factors for adverse events, we note that the treatment strategy influenced the prognosis in the medium term since the NO-CATH group was predictive of MACE, as were other clinically important factors such as Killip class and the presence of peripheral artery disease. Moreover, the NO-CATH group also showed a borderline statistically significant association with all-cause mortality.

### Limitations

Our study has several potential limitations inherent to the non-randomized observational design. Although registry studies often provide a more comprehensive picture of usual clinical practice, the included population tends to be heterogeneous, with diverse clinical characteristics what make difficult to draw generalized conclusions around certain therapeutic attitudes. Moreover, the inclusion criterion of “patients discharged with a diagnosis of NSTE-ACS” precluded consideration of people who died during their hospital stay. The fact that the patients included in our registry were admitted to hospitals equipped with a hemodynamic unit may have also introduced some selection bias, as these patients would have had easy and fast access to invasive care.

Furthermore, it is worth mentioning that the indication for different antiplatelets is not protocolized, rather, treatment was indicated by the attending physician. However, this can be considered a strength of the registry rather than a limitation, as it reflects usual practice for treating ACS, where many different professionals and services are involved in managing patients’ treatment.

Despite these limitations, we believe that our conclusions are of great interest because they derive from a voluntary registry where investigators’ role was limited to collecting data upon patient discharge. Thus, the performance of the study did not affect the clinical decisions made by attending physicians in any way. This voluntary character of the registry therefore ensures that the data are of excellent quality, a fact that has been corroborated by an independent external auditor.

## Conclusions

In this contemporary registry including patients with ACS, those NSTE-ACS patients who did not undergo catheterization during hospitalization represented a high-risk subgroup. They were undertreated with recommended therapies and showed lower prescription rates for NADs. The prognosis in the medium term was poor, with high mortality.
